# PLA/MWNTs Conductive Polymer Composites as Stress Sensors—The Role of Supramolecular Ordering

**DOI:** 10.3390/s26020414

**Published:** 2026-01-08

**Authors:** Łukasz Pietrzak, Michał Puchalski

**Affiliations:** 1 Institute of Mechatronics and Information Systems, Lodz University of Technology, 22 Bohdana Stefanowskiego Street, 90-537 Lodz, Poland; lukasz.pietrzak@p.lodz.pl; 2 Textile Institute, Lodz University of Technology, 116 Zeromskiego Street, 90-543 Lodz, Poland

**Keywords:** carbon nanotubes, nanocomposites, mechanical properties, electrical conductivity, stress sensors, supramolecular structure

## Abstract

**Highlights:**

**What are the main findings?**

- Developing PLA-based nanocomposite stress sensor with low MWNTs content.
- Identifying the limits of applicability of PLA/MWNTs stress sensors based on stress-induced crystallization phenomenon.

**What is the implication of the main finding?**

- A new approach to the development of resistive sensors based on the supramolecular ordering of PLA matrix.
- Validation of the relationship between the quantity of nanoaditive, the supramolecular structure of the polymer matrix, and the stability of the nanocomposite in assessing its sensory response to cyclic stress.

**Abstract:**

The incorporation of carbon nanostructures into polymer composites is of significant importance for the development of novel sensor materials, due to the excellent mechanical strength and variable electrical conductivity that these structures provide. It is evident that the significance of polylactide (PLA) and carbon nanotube (CNT) systems is attributable to two key factors. Firstly, these systems are notable for their environmental sustainability. Secondly, they exhibit enhanced functional properties. Despite the fact that a considerable number of studies have been conducted on conductive PLA/CNT composites, there has been limited research focusing on the supramolecular ordering of the polymer matrix and its impact on electromechanical properties. This factor, however, has been demonstrated in this study to significantly influence their response to applied stress and, consequently, their potential application as stress sensors. The present study has demonstrated that the precipitation method is an effective means of producing conductive PLA/MWNTs nanocomposites. This method is effective in ensuring the uniform dispersion of the filler in the polymer matrix, which creates an interesting prospect for mechanical sensors. It is evident that the durability of the nanocomposites is a key factor in ensuring the ordering of the supramolecular structure of the PLA matrix into the α form. The materials obtained were found to have a low percolation threshold of 0.2 wt.%. Furthermore, the practical application of these sensors, in the form of resistive strain sensors, was demonstrated for materials containing 5 wt.% of carbon nanotubes. The results presented here demonstrate that this methodology provides a novel perspective on the production of sensor materials, with the supramolecular ordering of the PLA matrix being a key factor.

## 1. Introduction

Conductive polymer composites (CPCs) are materials in which the polymer matrix, electrically insulating materials, is filled with conductive materials such as metal powder or nanomaterials, resulting in unique properties such as electrical conductivity [1,2,3]. For years, these materials have been of great significance in the development of new solutions for elastic electronics [4] or wearable electronics [5], known as textronics [6]. The formation of nanocomposites into a variety of structures is possible, including films [7], foams [8], fibers [9], and nonwovens [10]. The shape and macroscopic structure allow CPC materials to be applied very effectively not only as conductive elements but above all as sensors that react to changes in electrical conductivity under the influence of various external stimuli, such as volatile organic compounds (VOCs) [11], heat [12], or mechanical deformation [13].

The subject of CPC has been developed for years by many scientific centers around the world, and the research results confirm that composites can be made from practically any synthetic matrix. As demonstrated in the relevant literature, there are numerous examples of polymer matrices, including poly(methyl methacrylate) (PMMA) [14], polyvinyl alcohol (PVA) [15], polyacrylonitrile (PAN) [16], polyvinyl chloride (PVC) [17] polypropylene (PP) [18] and polylactide (PLA) [1]. The choice of matrix is important because it determines subsequent applications not only as an electrically conductive material but also mostly as a sensor. Interestingly, in the extensive literature on this subject matter, only a small number of publications discuss the aspect of the supramolecular structure of the polymer matrix, which, in accordance with the current state of knowledge, is of great importance in the case of PLA.

The physical and chemical properties of end products obtained from PLA are determined by a variety of stereoisomeric forms of lactide and the formation of different supramolecular structures of polymers. The properties of PLA are determined by the presence of units with different chirality in the polymer chain consisting of L and D isomers of lactide [19]. The presence of mixed chirality in PLA has been shown to reduce its crystallization potential, a factor that significantly affects the advantageous properties of the final product and its degradation rate [20]. The poly(l-lactide) homopolymer has the potential to crystallize in a variety of polymorphic ordered forms, including the pseudo-orthorhombic (a = 1.06 nm, b = 0.61 nm, c = 2.88, α = β = γ = 90°) and orthorhombic (a = 1.05 nm, b = 0.61 nm, c = 2.88 nm, α = β = γ = 90°) α form [21], the trigonal (a = 1.052 nm, b = 1.052 nm, c = 0.88 nm, α = β = 90°, γ = 120°) and the orthorhombic (a = 1.031 nm, b = 1.821 nm, c = 0.90 nm, α = β = γ = 90°) forms of β [22], orthorhombic (a = 0.995 nm, b = 0.625 nm, c = 0.88 nm, α = β = γ = 90°) γ form [23] or disordered and metastable pseudo-orthorhombic (a = 1.072 nm, b = 0.61 nm, c = 2.88 nm) α’ form [24]. Considering that the γ form is created through epitaxial crystallization and the β form by stretching the α form at a very high drawing ratio and high temperature, they are rarely observed in practical applications, including in CPCs. PLA products are therefore usually characterized by either an amorphous structure, an α’ form, or an α form crystalline structure. An interesting application and research aspect in the case of PLA is that of the crystallization and phase transition from disorder to order (α’ to α) on the thermomechanical process [25]. A particularly interesting research aspect is the in-depth study of the CPC material, given that the crystallization of the polymer matrix is induced by nuclei, which can be fillers, including nanostructures. This is especially pertinent in the context of semi-crystalline PLA [26].

PLA matrix could be filled with various types of materials to obtain CPC materials, among which are allotropic forms of carbon such as carbon black (CB) [27], carbon nanotubes (CNTs) [1,28] and graphene [29]. Despite the numerous advantages of various allotropic forms of carbon, two structures are of particular popularity: carbon black due to its production costs and carbon nanotubes, which, due to their properties [30], allow the development of composites with unique properties such as electroconductivity, tensile strength, and both electrostatic and electromagnetic shielding capabilities [28,31,32].

The incorporation of CNTs as an electrically conductive filler enables the creation of materials with interesting and applicable properties. However, achieving the desired functional properties in such nanocomposites necessitates the application of an appropriate manufacturing method. The primary challenge associated with the use of CNTs relates to their propensity to agglomerate into bundles, which are difficult to disperse. This phenomenon is attributable to van der Waals interactions between carbon nanotubes [33] and the remarkably elevated specific surface area of CNTs, which can attain several hundred square meters per gram [34,35].

It is evident that aggregation negatively impacts the optimization of filler dispersion, which is the process of determining the minimum additive content required to ensure the desired functional properties of the material. In the context of electrically conductive materials, the minimum amount of nano-additive is defined by the so-called percolation threshold [36]. The content in issue is determined by the configuration and dimensions of the nano-additives, in addition to their effective dispersion within the aggregating matrix [37]. The supramolecular structure of the polymer matrix is also an influencing factor, with the method of obtaining the composite having a significant influence on this structure [38]. Consequently, the process of obtaining the final product is complex, and the design of materials is multifaceted. In the case of PLA/MWNTs materials that are electrically sensitive to chemically positive stimuli, beneficial effects are achieved when the polymer matrix is amorphous. The omission of thermomechanical-induced crystallization, or the use of PLA with a high content of the D isomer, enables the formation of this material [10]. In contrast, there has been an increasing tendency for studies in the relevant literature to base the redistribution of the filler on what are termed segregated structures [26,39]. In these materials, percolation paths can be created both over greater distances than in a supramolecular structure [26] and using, for example, a spherulite structure in a polymer matrix [40]. Consequently, a wide range of possibilities is available, and, based on polymer physics, the material properties of CPCs can be designed.

In this paper, the authors present the results of research on the influence of the crystal structure of PLA on the properties of CPCs produced by precipitation. The proposed method was focused on minimizing the aggregation of nanotubes in the polymer matrix and the subsequent obtaining of composites with a low percolation threshold. The materials were characterized in depth using instrumental research methods. A range of studies were conducted using two analytical techniques, wide-angle X-ray diffraction (WAXD) and differential scanning calorimetry (DSC). These studies enabled the interpretation of the results of mechanical tests, electrical conductivity, and the sensitivity of the developed mechanical strain sensors. A comprehensive research approach confirmed that the design of CPC composites is a multifaceted issue and that supramolecular structure studies are necessary not only in the scientific sense but are also important for understanding the fundamentals of the obtained material properties.

## 2. Materials and Methods

### 2.1. Preparation of PLA/MWNTs Conductive Polymer Composites via Precipitation Method

The polylactide used as the polymer matrix was supplied by NatureWorks LLC (Plymouth, MN, USA), consisting of 97.5% l-lactide and 2.5% d-lactide. Analytical-grade trichloromethane (CHCl_3_), obtained from Chempur (Piekary Slaskie, Poland), served as the solvent for the PLA. This same solvent was also employed in preparing mixtures of multi-walled carbon nanotubes and PLA. The analytical-grade methyl alcohol used in the precipitation method was also provided by Chempur (Piekary Slaskie, Poland). The MWNTs used in this study were produced in our laboratory via the liquid source chemical vapor deposition (LSCVD) method [1,41]. The synthesis was carried out in a three-zone furnace, each zone equipped with its own temperature control, connected to a liquid catalyst delivery system and a supply of carrier gases. This setup enables precise adjustment of synthesis parameters—such as the temperature in each furnace zone, catalyst concentration, and carrier gas flow rate—allowing the properties of the resulting MWNTs, and consequently of the nanocomposite material, to be tuned [1,41]. Following each synthesis, the MWNTs deposits were analyzed using thermogravimetric analysis (TGA) and scanning electron microscopy (SEM) before proceeding to nanocomposite fabrication. The composite forming process involved creating a dispersion of nanotubes in trichloromethane in an ultrasonic bath for 1 h. The determined quantity of polymer granules is subsequently added to achieve the specified weight fraction in the final product. The polymer was continuously dissolved using ultrasonic dispersion for 90 min until a uniform solution was achieved by disaggregating the filler particle clusters created during manufacturing. The sonication duration for the CNT/solvent and CNT+PLA/solvent solutions was established through investigation conducted using light microscopy, subsequently followed by electron microscopy. Coagulation occurred in methanol, and the final structure of the composites was achieved through film pressing at 180 °C at a pressure of 500 kN.

### 2.2. TGA Method

The purity of synthesized MWNTs and the thermal stability of composites were carried out by means of thermogravimetry methods. Measurements were obtained by using the Q50 apparatus made by TA Instruments (New Castle, DE, USA), and the thermograph was recorded in the range of 50–850 °C with the step of 10 °C/min.

### 2.3. SEM Method

The micromorphology studies of obtained composites, as well as multiwalled carbon nanotubes, were performed using a scanning electron microscope, JEOL Ltd. IT200 (Akishima, Japan). The micrograph was recorded under a high vacuum regime by the detection of secondary electrons and electron beam energy at the level of 10 kV.

### 2.4. Mechanical Tests

Mechanical testing of obtained composites and stress sensing was performed by using a Linkam 200N Minitester (Salfords, Redhill, UK). To ensure full reproducibility of the results, five samples used for testing were prepared by punching them with a specialized template, guaranteeing identical shapes according to ASTM D638-22, Type IV standards [42]. The experiments were performed at room temperature—below the glass transition temperature (Tg) of polylactide with a deformation rate of 5% per minute.

### 2.5. Electroconductivity Tests

Electric conductivity of the PLA/MWNTs conductive polymer composites was examined using the four-electrode method, and in the case of electro-mechanical experiments, the two-electrode method was used. Highly conductive silver paste was used to create connections between the sample surface and the probes of digital multimeter Keithley 236 (Cleveland, OH, USA).

### 2.6. WAXD Supramolecular Structure Analysis

The supramolecular structures of the PLA samples were investigated by wide-angle X-ray diffraction (WAXD) using an X’Pert PRO diffractometer (CuKα source, λ = 0.154 nm) from PANalytical (Almelo, The Netherlands). The diffractograms for the powdered samples were recorded under I_a_ = 30 mA and U_p_ = 40 kV, over a 2θ range of 5° to 60° with a step of 0.05°.

### 2.7. Thermal Properties Analysis

The thermal properties of the PL/MWNTs composites were characterized using differential scanning calorimetry (DSC) with a Q2000 (TA Instruments, New Castle, DE, USA) apparatus. The specimens were initially heated from 0 °C to 250 °C, subsequently cooled back to 0 °C, and then immediately reheated to 250 °C at a rate of 10 °C/min. The measurements were obtained in a nitrogen atmosphere.

## 3. Results and Discussion

### 3.1. Electromechanical Sensor Investigations 

**Figure 1:**
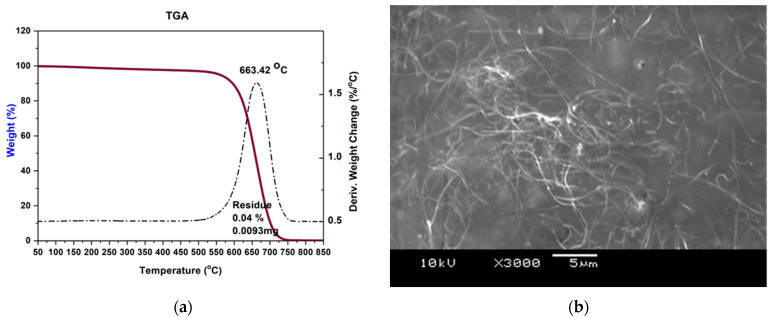
The results of quality assessment of MWNT and PLA/MWNT solution in chloroform: (**a**) TGA graph of synthesized MWNTs where the residue is only 0.04% by weight, (**b**) SEM image of homogeneous dispersion of carbon nanotubes in polylactide matrix.

In the preliminary phase of the research, the properties of the synthesized multi-walled carbon nanotubes and their solution in polymer were characterized. It is possible to synthesize correctly sized carbon nanotubes, with a high degree of purity, by appropriate adjustment of the synthesis parameters. In order to ensure the successful formation of an electrically conductive network in the polymer matrix, it is imperative to exercise precise control over the synthesis parameters, with particular attention to the dimensions of the filler particles, especially their length. Consequently, the synthesis stage significantly impacts the overall performance of the composite. The optimization of this process is of great significance for the purpose of obtaining high-purity materials, and this has the potential to eliminate the necessity for one or two purification steps and, moreover, to reduce the cost of the final product. The purity of the material was determined based on thermogravimetric analysis. As demonstrated in Figure 1a, the TG thermogram with the curve plotted indicates a single decomposition temperature of 663 °C, which is indicative of the decomposition of multi-walled carbon nanotubes (MWNTs). Following a comprehensive investigation of the nanotubes, the fabrication of polylactic acid nanocomposite samples continued using a consistent procedure for each batch of multi-walled carbon nanotube weight concentration. The selected production method for these nanocomposites is a solution-based procedure that entails the preparation of carbon nanotubes and the polymer for nanocomposite fabrication. The selection of trichloromethane was determined by its advantageous Hansen solubility properties for carbon nanotubes and its established efficacy as a solvent for the polylactic acid matrix utilized in this investigation [36]. These characteristics ensure a uniform distribution of the filler, as validated during the study process. Figure 1b demonstrates that the SEM image of the thin film derived from the CNT+PLA/solvent system verifies the uniform distribution of the nanofiller inside the polymer matrix, with few regions of aggregation. The existence of smaller carbon nanotube aggregates suggests that, even under optimal conditions, minor defects occur during the empirical process, potentially influencing subsequent outcomes. Additionally, SEM observations conducted under high vacuum environments demonstrated a distinct absence of charging effects on the surface of the nanocomposite film, signifying excellent electrical conductivity. This finding validates the establishment of an electrically conductive network of MWCNTs within a polylactide matrix.

**Figure 2:**
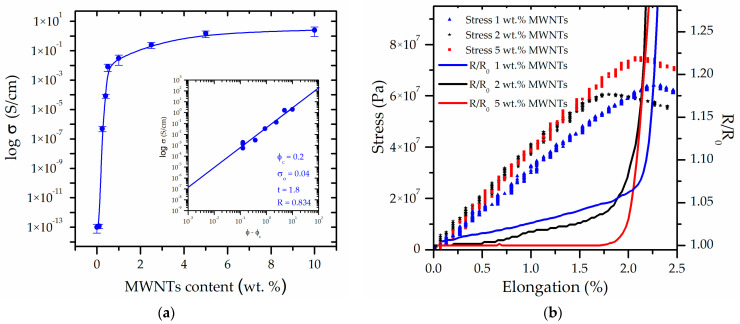
Electric properties of PLA/MWNTs composites: (**a**) electroconductivity as a function of filler concentration; error bars denote five times the standard deviations of nanocomposites; inset fitting of electric conductivities according to Equation (1); (**b**) stress-relative resistance (R/R_0_) as a function of elongation for MWNTs concentrations of 1 wt.%, 2 wt.%, and 5 wt.%.

In the initial phase of the investigation of composite materials, the electrical conductivity of nanocomposite materials was tested over a wide range of filler concentrations. The measurements were performed using a four-electrode method. The current-voltage characteristics of conductive polymer composites may include a linear region that directly corresponds to Ohm’s law, as well as a non-linear region that can be attributed, for example, to tunneling or doping effects occurring between filler particles separated by a distance of less than 10 nm [43]. In order to eliminate errors related to the non-linearity of the current-voltage characteristics and to ensure the highest possible repeatability and unambiguity of electrical conductivity measurements, the study focused on linear areas where Ohm’s law was satisfied. The uniformity of the experimental process was assured through the analysis of five samples, each characterized by a distinct specific weight fraction of filler. This methodology enabled the determination of the effect of the filler on electrical conductivity and the estimation of the percolation threshold. The electrical conductivity of the nanocomposite materials was investigated across a broad spectrum of filler concentrations, with the measured values ranging from 10^−13^ S/cm for the pure polymer to 2.7 S/cm at a 10 wt.% filler content (Figure 2a). The theoretical framework of percolation has been applied for an extended period in the analysis of the obtained results and the interpretation of the electrical conductivity of random mixtures of conductors and insulators [44]. In systems such as carbon nanotubes in a polylactide matrix, the volume conductivity increases gradually with increasing conductive filler below the percolation threshold. This phenomenon can be attributed to the principles of quantum mechanics, which state that the increase in conductivity is due to the increased tendency of electrons to effectively tunnel between insulated conductive fillers as the distance between them decreases. When the conductive filler content exceeds the threshold, a significant increase in conductivity is observed, as demonstrated in Figure 2a. The change in conductivity (*σ*) above the percolation threshold can be expressed in the following form, as developed by Kirkpatrick [43], Stauffer and Aharony [45]:

(1) $$\sigma =\sigma_{0}{\left(\phi -\phi_{c}\right)}^{t},\;\;\phi >\phi_{c}$$

where *ϕ* is the weight fraction, *ϕ_c_* is the weight fraction percolation threshold or threshold probability of formation of a conducting network, and *σ*_0_ and t denote the proportionality coefficient and the exponent of the weight fractions percolation model, respectively. The most significant finding of the study is that the examination of samples obtained using the precipitation method demonstrates a remarkably low percolation threshold of 0.2 wt.% and a critical exponent of 1.8.

The conductivity values near the percolation threshold parameters ensure effective shielding against static electricity in nanocomposites. The low percolation makes it possible to lower the production costs of protective layers or parts of sensors based on the conductivity change in function of the applied stress. 

Prior to analyzing the relationship between stress and electrical conductivity of the analyzed material, the essential mechanical properties were methodically investigated. This preliminary step was necessary in order to establish the most suitable MWNTs content that would ensure not only reliable sensor functionality but also sufficient structural integrity of the composite matrix. In particular, the focus was placed on two key parameters: Young’s modulus, which reflects the stiffness of the material, and the tensile yield strength, which indicates the onset of permanent deformation under applied stress. Both properties were measured across a broad range of filler contents, allowing for a comprehensive assessment of how the increasing MWNTs concentration influenced the overall mechanical response of the system. The analyzed mechanical properties of the nanocomposites demonstrated exceptional consistency, with variations in the obtained values not above 10 wt.%. Table 1 summarizes the mechanical characterization data, illustrating the relationship between Young’s modulus and tensile yield strength in response to the MWNTs filler concentration. These results establish a baseline for a further investigation of the relationship between stress and electrical conductivity, thereby confirming that the selected MWNTs concentrations are appropriate for the fabrication of efficient stress-sensitive composite sensors (Figure 2b). It is important to note that increasing the concentration of carbon nanotubes from 1 wt.% to 2 wt.% enhances the Young’s modulus and yield strength, but this impact is not evident in terms of elongation and tenacity, which instead exhibit an insignificant decrease. This could result from the redistribution of carbon nanotubes, which, as evidenced by SEM data, can aggregate locally. A noticeable change in characteristics is evident even at a concentration of 5 wt.%, with no change in relative resistivity observed up to 1.5% strain. This is a significant finding for the selection of concentration for the proposed application.

As demonstrated in Table 1, the incorporation of multi-walled carbon nanotubes into the polylactide matrix impacts the mechanical properties of composites, with varying degrees of influence depending on the specific property under consideration.

In relation to Young’s modulus, the incorporation of MWNT contents as low as 0.15% and 0.25% by weight exhibited only marginal enhancements in comparison to pure PLA, with no substantial deviations being observed within the experimental margin of error (2.77 GPa for pure PLA compared to 2.79 GPa at 0.15% by weight). A significant enhancement in stiffness was evident only at carbon nanotube contents exceeding 0.25% by weight, with the modulus attaining 3.31 GPa at 0.25% by weight and progressively escalating to 3.26 GPa at 5% by weight. This non-linear behavior suggests that at low concentrations of carbon nanotubes, they may not form sufficient load-bearing networks in the polymer matrix, thus limiting their reinforcing effectiveness. However, at concentrations that exceed the specified threshold (ranging from 0.25 to 0.5% by weight), the nanotubes appear to make a substantial contribution to the stress transfer mechanism, thereby increasing the stiffness of the composite material. Such a sharp increase in modulus at higher CNT contents is consistent with the emergence of a filler network that effectively restricts the mobility of polymer chains, thereby increasing stiffness.

A similar general trend was observed for tensile strength, although the improvement was more gradual and continuous compared to the sharp changes in the modulus of elasticity. The tensile strength of pure PLA was measured at 51.3 MPa, and the addition of 0.15% by weight of MWNTs resulted in only a slight increase to 51.4 MPa. A more pronounced improvement was observed with further increases in MWNTs content, reaching 54.1 MPa at 0.25% by weight and 54.6 MPa at 0.5% by weight. The tensile strength of the material was found to reach a peak value of 58.7 MPa at 1% by weight of carbon nanotubes, thus demonstrating a significant reinforcement in comparison to pure polymer. It is interesting to note that at 2 wt.% content, a slight decrease to 57.8 MPa was observed. This can be attributed to the tendency of carbon nanotubes to agglomerate at higher concentrations or the initialization of local order supramolecular structure, causing localized stress concentrations that reduce the overall strength. However, at the highest load tested (5% by weight), a significant increase in tensile strength was observed, reaching 65.9 MPa. This finding indicates that, at sufficiently high filler content, the reinforcing effect of carbon nanotubes may potentially overcome the adverse effects of agglomeration.

In summary, these results show that reinforcing PLA with carbon nanotubes has a dual effect: while stiffness (Young’s modulus) increases significantly after reaching a critical filler content, tensile strength exhibits a more complex relationship, characterized by initial reinforcement, temporary decrease, and then a return to normal at higher loads. This behavior highlights the importance of balancing carbon nanotube concentration, dispersion quality, and interfacial adhesion, as well as the supramolecular structure of the matrix when designing nanocomposites for stress detection applications. From a practical standpoint, these results suggest that carbon nanotube concentrations in the range of 1–2% by weight may represent an optimal compromise between mechanical reinforcement and processability while providing sufficient electrical conductivity for sensor applications.

Figure 2b shows the results for stress-relative resistance (R/R_0_) for MWNTs concentrations of 1 wt.%, 2 wt.% and 5 wt.%. The results obtained from the sample containing 1 wt.% of MWNTs demonstrate that changes in resistivity occur across nearly the entire strain range. This finding indicates a high sensitivity of the conductive network even to minor deformations. In contrast, for the composite with 2 wt.% MWNTs, noticeable changes emerge only after approximately 0.25% strain, suggesting a more stable percolated network that requires a threshold deformation to disturb electron transport pathways. The most striking effect is observed in the sample with the highest filler concentration investigated (5 wt.%), where no discernible change in resistivity is observed up to ~1.75% strain. This behavior suggests that, at elevated nanotube loading, the conductive network becomes so dense and redundant that minor deformations are inadequate to disrupt percolation.

It is crucial to highlight the relationship between the amount of deformation and the extent of change in resistivity. It was demonstrated that up to the elasticity limit, the increase in relative resistivity for the 1% by weight sample did not exceed 8%, while in the 2% by weight sample, this increase was less than 5%. In the case of the 5% by weight sample, practically no changes in the elasticity range were observed. It is evident that a distinct and swift rise in electrical resistance occurs exclusively once the yield point has been exceeded in all the examined nanocomposites. This rapid increase is attributed to the progressive destruction of the conductive pathways, specifically the loss of contacts between the nanotubes in the percolation network structure. It is noteworthy that the tensile yield strength is determined by the quantity of additive and the potential ordering in the supramolecular structure of PLA.

This study provides a mechanistic perspective, demonstrating that the filler concentration exerts a substantial influence on the electromechanical response of MWNTs/polymer nanocomposites. At lower concentrations, the conductive network is sparse and more brittle, leading to continuous sensitivity to deformation. As the filler content increases, the conductive network becomes more robust and less sensitive to minor deformations. However, once the elasticity limit is exceeded, a catastrophic loss of conductive connections causes a dramatic increase in resistance. This underscores the dual function of the filler fraction, which serves to enhance the electrical stability of the composite under minor deformations while concurrently determining the deformation threshold beyond which the conductive network undergoes irreversible structural degradation.

**Figure 3:**
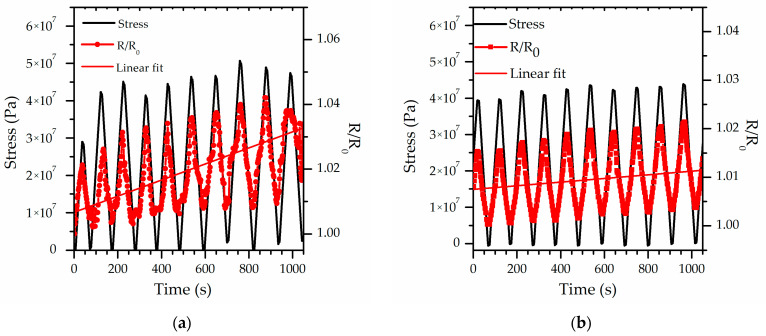
Relative resistance (R/R_0_) as a function of applied stress: (**a**) results for composite containing 1 wt.% of MWNTs, (**b**) results for the composite containing 5 wt.% of MWNTs.

The initial study results allowed for the selection of a concentration of carbon nanotubes that is both useful and suitable for additional in-depth analysis. As illustrated in Figure 3, the results of the sensitivity of composites as changes in relative resistance under cyclic stresses are presented. The experiment was conducted on samples containing 1 wt.% and 5 wt.% of carbon nanotubes, which enabled an additional comparison of the effect of filler content on the electromechanical response. 

In the case of both graphs, two key observations can be clearly identified. The initial observation relates to the absence of any significant change in relative resistance with respect to the initial values at the onset of the experiment, a finding that holds true for both the 1 wt.% and the 5 wt.% CNT composites. The effect is particularly evident when considering the minima of the resistance curves, which consistently occur at points where the applied stress is either zero or close to zero. The second is an increase in relative resistance after cyclic stressing of the sample. In the case of a composite containing 1% by weight of carbon nanotubes, the relative increase in resistance is slightly over 1.5% after 10 cycles of stressing and unstressing. In contrast, for a sample containing 5% by weight of carbon nanotubes, this increase is only about 0.5% after 10 cycles. These relatively small changes suggest a high degree of structural stability of the conductive network under cyclic loading, at least within the tested strain range. This phenomenon is based on the described results of the relative resistance changes in the tensile test. It also confirms that using a higher concentration of nanoadditives is more beneficial as it provides more stable sensory properties for the conductive polymer composite. However, in the case of the 1 wt.% sample, an additional phenomenon becomes noticeable after the tenth cycle. In this experiment, the resistance curve exhibited a deviation that appeared to be attributable not only to the intrinsic response of the material but also to other factors. Conversely, this phenomenon is associated with an increase in electrical contact resistance between the specimen and the measuring apparatus. It is imperative to acknowledge that such behavior underscores the necessity of incorporating not only the intrinsic piezoresistive properties of the composite into the analysis but also the stability of the electrode-sample interfaces when interpreting long-term cycling measurements.

### 3.2. Supramolecular Structural Study of PLA/MWNTs Conductive Polymer Composites

**Figure 4:**
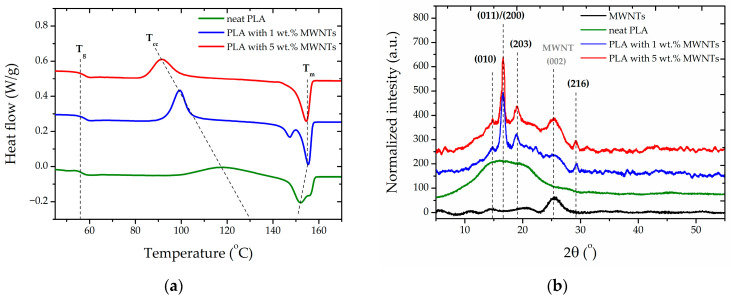
DSC thermograms (**a**) and WAXD profiles (**b**) of the studied materials.

In order to understand the observed phenomena in greater depth, additional structural investigations of polylactide were conducted. Initially, phase transitions were evaluated using DSC. As illustrated in Figure 4b, the incorporation of a nano-additive into the polymer structure appears to have a significant effect on phase transitions. Despite the negligible change in glass transition point and the temperature and ΔCp, which are 56.6 °C and around 1.5 J/(g·°C), respectively, cold crystallization is evidently influenced by the addition of carbon nanotubes. It has been demonstrated that an increase in the nano additive content results in a decrease in the cold crystallization temperature. Moreover, the temperature range within which this phenomenon occurs is narrower in comparison with the unmodified polymer, where cold crystallization occurs practically until the onset of melting. The enthalpy change values for cold crystallization are comparable, with measured values of 17.5 J/g, 19.4 J/g and 20.1 J/g for neat PLA, PLA with 1 wt.% MWNTs, and PLA with 5 wt.% MWNT, respectively. The slight rise in ΔH_cc_ values may be attributable to the rapid nucleation of the crystalline structure prior to the addition of the MWNTs, which also corresponds to the observation of this phenomenon at a reduced temperature with a considerably more narrow peak on the thermogram. Furthermore, notable changes in the melting point were observed upon the addition of MNWT to the polyacetal matrix. It is evident that the incorporation of carbon nanotubes leads to an increase in the maximum melting point from 152 °C for neat PLA to 156 °C for PLA with 5 wt.% MWNTs added. This is in addition to the other changes in properties that have been observed. The melting point for neat PLA evidently exhibits a secondary peak at 155°, indicative of the fact that the method of preparation promotes the crystallization of PLA into two similar crystalline forms. Two melting peaks are also visible for the composite with 1 wt.% MWNTs, but the one at the higher temperature is dominant. A comparative analysis of the enthalpy values at the melting point reveals a similarity with the enthalpy at cold crystallinity. This finding suggests that the materials are amorphous, with a small percentage of crystallite content. It is reasonable to hypothesize, based on the results obtained, that neat PLA crystallizes into a disordered α’ form. However, with an increase in MWNTs content, the material shows the ability to crystallize directly into a stable, ordered α form. This observation is significant and serves as a basis for a more comprehensive evaluation by means of X-ray diffraction.

In Figure 4b, X-ray diffraction profiles recorded for PLA/MNWTs composites with comparison to neat PLA and carbon nanotubes are presented. The crystalline structure of multiwall carbon nanotubes is represented by the rolled multilayer of graphene and visible by wide peaks around 2θ value 25°. However, in the case of pure PLA, only a broad halo typical of the amorphous structure of PLLA is visible. 

As illustrated in Figure 4b, the X-ray diffraction profiles obtained for the PLA/MNWT composites are presented in comparison to both neat PLA and carbon nanotubes. The crystalline structure of multiwall carbon nanotubes is represented by rolled multilayer graphene, which has a graphite-like structure. This is visible by a wide peak around the 2θ value of 25°. In the case of neat PLA, a broad halo is observed, which is characteristic of the amorphous structure of PLLA. 

The WAXD profiles for the materials under investigation demonstrate significant differences. The presence of carbon nanotubes is indicated by the presence of a peak at approximately 2θ value 25°, the intensity of which increases with the percentage of MWNT, as would be expected. In addition to the amorphous halo, X-ray diffraction peaks are visible at 2θ values of 14.7°, 16.8°, 18.9°, and 29.1°, corresponding to the (010), (011)/(200), (203), and (216) lattice planes of the PLLA order α form, respectively. The presence of visible diffraction peaks, notably corresponding to the (010) and (216) lattice planes, confirms the direct crystallization of polylactide into a highly ordered α-form structure during the composite production process. This is a consequence of the nucleation of the structure, as supported by nanoadditives. This conclusion is supported by DSC thermograms in which a marked increase in melting point temperature was observed. 

A more comprehensive numerical analysis of X-ray diffraction profiles was conducted using the WAXSFIT 1.0 software based on Hindeleh and Johnson’s method [46,47]. Deconvolution of the crystalline and amorphous halo components indicates that the crystallinity level is 15.6% and 17.4% for composites containing 1 wt.% and 5 wt.% MWNTs, respectively. Furthermore, for the most pronounced peaks at angles of 16.9 and 18.9, corresponding to the (011)/(200) and (203) lattice planes, the parameters d-spacing and the average crystallite size were estimated based on the Bragg’s equation [48] and the Scherrer equation [49,50], respectively. For both materials studied, the distances were d_(011)/(200)_ = 0.519 nm and d_(203)_ = 0.471 nm, thereby confirming the high structural order. Conversely, the average dimensions of the crystalline regions at the L_(011)/(200)_ = 11.6 nm and L_(203)_ = 11.2 nm levels suggest the presence of comparatively substantial crystalline domains within the polymer material. The values of the structural parameters enable the mechanical properties of the composites to be interpreted in terms of supramolecular changes. The presence of a small amount of highly ordered structure is evidenced by the high values of Young’s modulus and plasticity.

Moreover, the supramolecular order of the polymer matrix is advantageous in the context of designing a stress sensor material. Cyclic stresses have been shown in experimental studies of this material to have no significant effect on relative resistance. This is explained by the findings that the supramolecular structure of the matrix is stable and that this is the reason for the lack of deterioration.

## 4. Conclusions

The outcomes of the investigations demonstrated a successful creation of electrically conductive PLA/MWNTs nanocomposites by the method of precipitation. The applied approach guaranteed sufficient dispersion of carbon nanotubes within the polymer matrix and allowed the formation of an ordered α crystalline structure of PLA, resulting in materials with notable features, including functionality as a mechanical strain sensor. The presence of an ordered crystalline structure improves the elasticity of the polymer matrix. Furthermore, it eliminates the possibility of a disorder-to-order (α’ to α) phase transition during application, which might negatively impact the percolation pathways. A notable advantage is that the resulting material will degrade more slowly than if it were amorphous, as confirmed by the current state of knowledge about PLA. The mechanical characterization of the composites revealed that the addition of carbon nanotubes had a significant impact on both the stiffness and tensile strength of the composites. The Young’s modulus demonstrated minimal change at low filler contents but increased substantially above 0.25–0.5 wt.%, reaching up to 3.26 GPa at 5 wt.%. Tensile strength showed a more complex trend: a steady increase up to 1 wt.% CNTs, a slight decline at 2 wt.% due to potential agglomeration effects, and a significant recovery at 5 wt.%. The results of this study highlight the importance of identifying optimal MWNTs concentrations that balance reinforcement, processability, and dispersion quality.

Electrical conductivity measurements confirmed the creation of a percolating network at very low filler contents, with a percolation threshold as low as 0.2 wt.%. The ability to achieve conductivity at such low loads highlights the efficiency of the proposed manufacturing technique. However, it is worth noting that stable sensors require filling with at least 1 wt.%, and preferably 5 wt.%, which shows the difference between creating a conductive material compared to one with a sensing function. Electromechanical testing demonstrated that the composites exhibit strain- and stress-dependent changes in electrical resistance, thus confirming their suitability for stress-sensing applications. In the case of low MWNTs contents (1 wt.%), the conductive network demonstrated a high degree of sensitivity to deformation, resulting in gradual resistance changes even within the elastic regime. As the filler concentration increased, the conductive pathways became more robust, delaying the onset of resistivity changes until larger deformations had occurred. At a concentration of 5 wt.%, the network demonstrated stability under low levels of strain but, upon surpassing the yield point, a pronounced increase in resistance was observed, indicative of the breakdown of conductive junctions. Furthermore, the results of the cyclic loading experiments demonstrated the excellent stability of the electrical response, with only minor baseline drifts being observed after repeated cycles.

The investigation of the supramolecular structure resulted in a more insightful perspective on the obtained outcomes. The postulated statement regarding the significance of high redistribution of the nanoadditive should be extended, based on the obtained DSC and WAXD results, to include the importance of supramolecular structure ordering. The incorporation of MWNTs has been demonstrated to induce nucleation and direct the crystallization of PLA into the ordered α form, a phenomenon that is not typically observed during the processing of this polymer. The crystalline phase content is low (up to 17.5%) in semi-crystalline PLA but the quality of it significantly determines the mechanical characteristics and cycle durability of the sensors. The order α form, detectable at a 1% weight content of carbon nanotubes, improves the material’s stability as a sensor during cyclic deformation, which is particularly noticeable at 5% weight.

## Figures and Tables

**Table 1:** Mean values of Young’s modulus and tensile yield strength for varying concentrations of MWNTs.

Filler Weight Content (%)	Young’s Modulus (GPa)	Tensile Yield Strength (MPa)
0 (neat PLA)	2.77 (0.27) *	51.3 (4.88)
0.15	2.79 (0.26)	51.4 (4.98)
0.25	3.31 (0.29)	54.1 (4.87)
0.5	3.33 (0.31)	54.6 (4.92)
1	3.22 (0.30)	58.7 (4.87)
2	3.26 (0.31)	57.8 (4.98)
5	3.26 (0.29)	65.9 (5.56)

* The standard deviation is in brackets.

## Data Availability

The original contributions presented in this study are included in the article. Further inquiries can be directed to the corresponding author.

## Abbreviations

The following abbreviations are used in this manuscript:

MWNTs	Multiwall carbon nanotubes
CPCs	Conductive Polymer Composites
PLA	Polylactide
PLLA	Poly(l-lactide)
SEM	Scanning Electron Microscopy
TGA	Thermogravimetric Analysis
DSC	Dynamic Scanning Calorimetry
WAXD	Wide-angle X-ray diffractometer

## References

[1] Pietrzak L., Raniszewski G., Szymanski L. (2022). Multiwalled Carbon Nanotubes Polylactide Composites for Electrical Engineering—Fabrication and Electrical Properties. Electronics.

[2] Zhang W., Dehghani-Sanij A.A., Blackburn R.S. (2007). Carbon Based Conductive Polymer Composites. J. Mater. Sci..

[3] Badrul F., Halim K.A.A., Salleh M.A.A.M., Omar M.F., Osman A.F., Zakaria M.S. (2019). Current Advancement in Electrically Conductive Polymer Composites for Electronic Interconnect Applications: A Short Review. IOP Conf. Ser. Mater. Sci. Eng..

[4] Liu H., Li Q., Zhang S., Yin R., Liu X., He Y., Dai K., Shan C., Guo J., Liu C. (2018). Electrically Conductive Polymer Composites for Smart Flexible Strain Sensors: A Critical Review. J. Mater. Chem. C Mater..

[5] Tang W., Sun Q., Wang Z.L. (2023). Self-Powered Sensing in Wearable Electronics—A Paradigm Shift Technology. Chem. Rev..

[6] Gniotek K., Krucińska I. (2004). The Basic Problems of Textronics. Fibres Text. East. Eur..

[7] Satapathy B.K., Weidisch R., Pötschke P., Janke A. (2005). Crack Toughness Behaviour of Multiwalled Carbon Nanotube (MWNT)/Polycarbonate Nanocomposites. Macromol. Rapid Commun..

[8] Baltopoulos A., Athanasopoulos N., Fotiou I., Vavouliotis A., Kostopoulos V. (2013). Sensing Strain and Damage in Polyurethane-MWCNT Nano-Composite Foams Using Electrical Measurements. Express Polym. Lett..

[9] Pötschke P., Andres T., Villmow T., Pegel S., Brünig H., Kobashi K., Fischer D., Häussler L. (2010). Liquid Sensing Properties of Fibres Prepared by Melt Spinning from Poly(Lactic Acid) Containing Multi-Walled Carbon Nanotubes. Compos. Sci. Technol..

[10] Krucińska I., Surma B., Chrzanowski M., Skrzetuska E., Puchalski M. (2013). Application of melt-blown technology in the manufacturing of a solvent vapor-sensitive, non-woven fabric composed of poly(lactic acid) loaded with multi-walled carbon nanotubes. Text. Res. J..

[11] Krucińska I., Surma B., Chrzanowski M., Skrzetuska E., Puchalski M. (2013). Application of Melt-blown Technology for the Manufacture of Temperature-sensitive Nonwoven Fabrics Composed of Polymer Blends PP/PCL Loaded with Multiwall Carbon Nanotubes. J. Appl. Polym. Sci..

[12] Balam A., Cen-Puc M., May-Pat A., Abot J.L., Avilés F. (2019). Influence of Polymer Matrix on the Sensing Capabilities of Carbon Nanotube Polymeric Thermistors. Smart Mater. Struct..

[13] Flandin L., Bréchet Y., Cavaillé J.Y. (2001). Electrically Conductive Polymer Nanocomposites as Deformation Sensors. Compos. Sci. Technol..

[14] Stéphan C. (2000). Electrical Properties of Singlewalled Carbon Nanotubes-PMMA Composites. AIP Conf. Proc..

[15] Xue P., Tao X.M., Park K.H. (2011). Electrically Conductive Fibers/Yarns with Sensing Behavior from PVA and Carbon Black. Key Eng. Mater..

[16] Mikołajczyk T., Rabiej S., Szparaga G., Boguń M., Fraczek-Szczypta A., Błażewicz S. (2009). Strength Properties of Polyacrylonitrile (PAN) Fibres Modified with Carbon Nanotubes with Respect to Their Porous and Supramolecular Structure. Fibres Text. East. Eur..

[17] Mamunya Y., Boudenne A., Lebovka N., Ibos L., Candau Y., Lisunova M. (2008). Electrical and Thermophysical Behaviour of PVC-MWCNT Nanocomposites. Compos. Sci. Technol..

[18] Al-Saleh M.H. (2015). Electrically Conductive Carbon Nanotube/Polypropylene Nanocomposite with Improved Mechanical Properties. Mater. Des..

[19] Saeidlou S., Huneault M.A., Li H., Park C.B. (2012). Poly (Lactic Acid) Crystallization. Prog. Polym. Sci..

[20] Giełdowska M., Puchalski M., Sztajnowski S., Krucińska I. (2022). Evolution of the Molecular and Supramolecular Structures of PLA during the Thermally Supported Hydrolytic Degradation of Wet Spinning Fibers. Macromolecules.

[21] Kawai T., Rahman N., Matsuba G., Nishida K., Kanaya T., Nakano M., Okamoto H., Kawada J., Usuki A., Honma N. (2007). Crystallization and Melting Behavior of Poly (L-Lactic Acid). Macromolecules.

[22] Kazuyo D.S., Aki Sasashige T., Kanamoto T., Hyon S.H. (2003). Preparation of Oriented β-Form Poly(l-Lactic Acid) by Solid-State Coextrusion: Effect of Extrusion Variables. Macromolecules.

[23] Cartier L., Okihara T., Ikada Y., Tsuji H., Puiggali J., Lotz B. (2000). Epitaxial Crystallization and Crystalline Polymorphism of Polylactides. Polymer.

[24] Pan P., Kai W., Zhu B., Dong T., Inoue Y. (2007). Polymorphous crystallization and multiple melting behavior of poly(L-lactide): Molecular weight dependence. Macromolecules.

[25] Stoclet G., Seguela R., Vanmansart C., Rochas C., Lefebvre J.-M. (2012). WAXS study of the structural reorganization of semi-crystalline polylactide under tensile drawing. Polymer.

[26] Masiuchok O., Iurzhenko M., Kolisnyk R., Mamunya Y., Godzierz M., Demchenko V., Yermolenko D., Shadrin A. (2022). Polylactide/Carbon Black Segregated Composites for 3D Printing of Conductive Products. Polymers.

[27] Narkis M., Ram A., Flashner F. (1978). Electrical Properties of Carbon Black Filled Polyethylene. Polym. Eng. Sci..

[28] Pietrzak Ł., Stano E., Szymański Ł. (2024). The Electromagnetic Shielding Properties of Biodegradable Carbon Nanotube–Polymer Composites. Electronics.

[29] Gu T., Zeng Z., Wu S., Sun D.X., Zhao C.S., Wang Y. (2023). Poly (L-Lactic Acid)/Graphene Composite Films with Asymmetric Sandwich Structure for Thermal Management and Electromagnetic Interference Shielding. Chem. Eng. J..

[30] Bernholc J., Brenner D., Buongiorno Nardelli M., Meunier V., Roland C. (2002). Mechanical and Electrical Properties of Nanotubes. Annu. Rev. Mater. Sci..

[31] Thostenson E., Ren Z., Chou T.-W. (2001). Advances in the Science and Technology of Carbon Nanotubes and Their Composites: A Review. Compos. Sci. Technol..

[32] Suckeveriene R.Y., Zelikman E., Narkis M. (2011). Hybrid Electrically Conducting Nano Composites Comprising Carbon Nanotubes/Intrinsically Conducting Polymer Systems. Wiley Encyclopedia of Composites.

[33] Sun C.H., Lu G.Q., Cheng H.M. (2006). Simple Approach to Estimating the van Der Waals Interaction between Carbon Nanotubes. Phys. Rev. B.

[34] Peigney A., Laurent C., Flahaut E., Bacsa R., Rousset A., Peigney A., Laurent C., Flahaut E., Bacsa R., Rousset A. (2001). Specific Surface Area of Carbon Nanotubes and Bundles of Carbon Nanotubes. Carbon.

[35] Lehman J.H., Terrones M., Mansfield E., Hurst K.E., Meunier V. (2011). Evaluating the Characteristics of Multiwall Carbon Nanotubes. Carbon.

[36] Gårdebjer S., Andersson M., Engström J., Restorp P., Persson M., Larsson A. (2016). Using Hansen Solubility Parameters to Predict the Dispersion of Nano-Particles in Polymeric Films. Polym. Chem..

[37] Li J., Kim J.K. (2007). Percolation Threshold of Conducting Polymer Composites Containing 3D Randomly Distributed Graphite Nanoplatelets. Compos. Sci. Technol..

[38] Sobkowicz M.J., White E.A., Dorgan J.R. (2011). Supramolecular Bionanocomposites 3: Effects of Surface Functionality on Electrical and Mechanical Percolation. J. Appl. Polym. Sci..

[39] Pang H., Xu L., Yan D.-X., Li Z.-M. (2014). Conductive polymer composites with segregated structures. Prog. Polym. Sci..

[40] de Souza Vieira L., dos Anjos E.G.R., Verginio G.E.A., Oyama I.C., Braga N.F., da Silva T.F., Montagna L.S., Passador F.R. (2021). A review concerning the main factors that interfere in the electrical percolation threshold content of polymeric antistatic packaging with carbon fillers as antistatic agent. Nano Select..

[41] Raniszewski G., Pietrzak Ł. (2021). Optimization of Mass Flow in the Synthesis of Ferromagnetic Carbon Nanotubes in Chemical Vapor Deposition System. Materials.

[42] (2022). Standard Test Method for Tensile Properties of Plastics.

[43] Koncar V. (2018). Smart Textiles for In Situ Monitoring of Composites.

[44] Kirkpatrick S. (1973). Percolation and Conduction. Rev. Mod. Phys..

[45] Stauffer D., Aharony A. (1994). Introduction to Percolation Theory.

[46] Rabiej M. (2013). Application of Immune and Genetic Algorithms to the Identification of a Polymer Based on Its X-Ray Diffraction Curve. J. Appl. Crystallogr..

[47] Hindeleh A.M., Johnson D.J. (1974). Crystallinity and Crystallite Size Measurement in Cellulose Fibres: 2. Viscose Rayon. Polymer.

[48] Lawrence Bragg—Nobel Lecture. NobelPrize.Org. https://www.nobelprize.org/prizes/physics/1915/wl-bragg/lecture/.

[49] Scherrer P. (1918). Nachrichten von der Gesellschaft der Wissenschaften zu Göttingen, Mathematisch-Physikalische Klasse.

[50] Holzwarth U., Gibson N. (2011). The Scherrer Equation versus the “Debye-Scherrer Equation”. Nat. Nanotechnol..

